# Multiple lymphomatous polyposis: A presentation for mantle cell lymphoma

**DOI:** 10.1002/ccr3.3291

**Published:** 2020-09-17

**Authors:** Cory Gallivan, Ali Akbar

**Affiliations:** ^1^ Burrell College of Osteopathic Medicine Roswell New Mexico USA

**Keywords:** mantle cell lymphoma, polyposis

## Abstract

Multiple lymphomatous polyposis is an uncommon presentation of mantle cell lymphoma, and when exhibited should prompt clinicians to incorporate mantle cell lymphoma into their differential workup.

## Graphical abstract

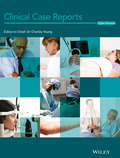

## 1 CASE DESCRIPTION

A 78‐year‐old woman was presented to the hospital without prior colorectal cancer screening. Physical examination was significant for Virchow's node (Troisier's sign). Colonoscopy revealed innumerable uniform polyps most heavily concentrated in the cecum. Biopsy revealed mantle cell lymphoma. A 78‐year‐old woman with history of hypertension and hyperlipidemia presents to the surgical clinic for colonoscopy. Upon review of systems, patient reports dysphagia. Her physical examination was remarkable for a soft nontender anterior cervical lymphadenopathy, and a left supraclavicular lymph node enlargement (Virchow's node; Troisier's sign). Upper endoscopy and colonoscopy were scheduled within the next week. Upper endoscopy revealed a partially occluding oropharyngeal submucosal lesion. There were no signs of ulceration or any mucosal involvement. Some polyps were observed within the esophagus, but the stomach and duodenum were unremarkable. Colonoscopy revealed numerous polyps were increasing in quantity from the transverse colon to the cecum. On colonoscopic inspection, polyps were uniform in size and shape resembling a polyposis syndrome. Multiple biopsies were obtained in the cecum, and ascending and transverse colon, and then sent to pathology. Initial pathology report returned with the polyps as atypical lymphocytic infiltrate, which was later identified to be mantle cell lymphoma. Outside of the lymphatic system, mantle cell lymphoma is seen as a multiple lymphomatous polyposis, or lymphomatous intestinal polyposis. Its detection and identification are of utmost importance due to the aggressive nature of the disease. This is a patient who presented with a physical examination concerning for malignancy, which was later confirmed via biopsies in the colon and in a cervical lymph node. Mantle cell lymphoma is known as a late‐presenting aggressive cancer. Outside of the lymphatic system, it can present as lymphomatous intestinal polyposis or multiple lymphomatous polyposis in the colon. This phenomenon should be recognized and incorporated into the differential workup of polypoid diseases of the colon.

**Figure 1:**
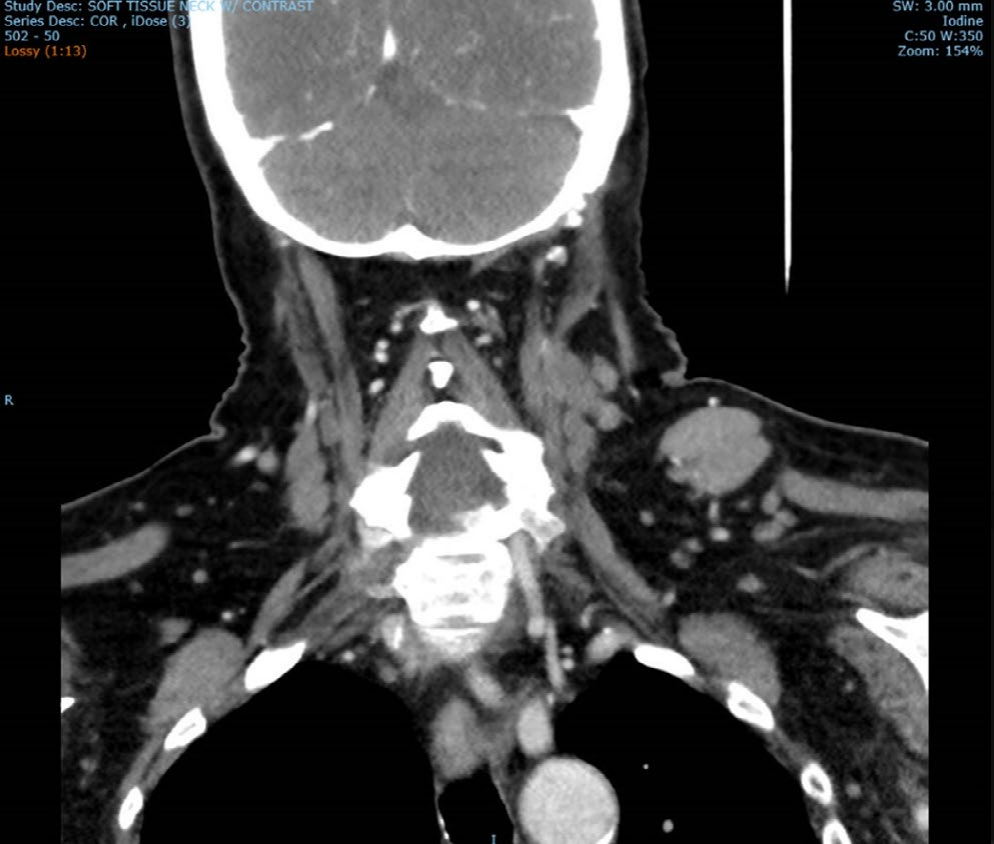
Coronal CT soft tissue Virchow's node

**Figure 2:**
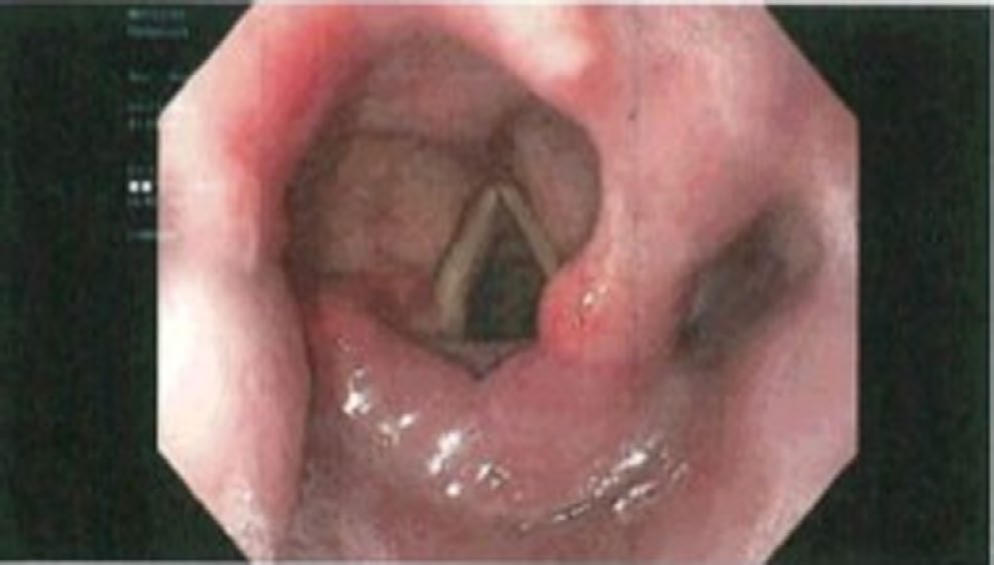
Endoscopy of laryngeal mass

A 78‐year‐old woman with past medical history significant for hypertension and hyperlipidemia presents to the surgical clinic for colonoscopy. The patient denied history of a colorectal screening examination. Upon review of systems, the patient reported dysphagia. Her physical examination was remarkable for a soft nontender anterior cervical lymphadenopathy and a left supraclavicular lymph node enlargement (Troisier's sign; Virchow's node; Figure 1). Due to the concerning physical examination findings, upper endoscopy and colonoscopy were scheduled within the next week. Upper endoscopy revealed an oropharyngeal submucosal lesion in the lumen as seen in Figure 2.

**Figure 3:**
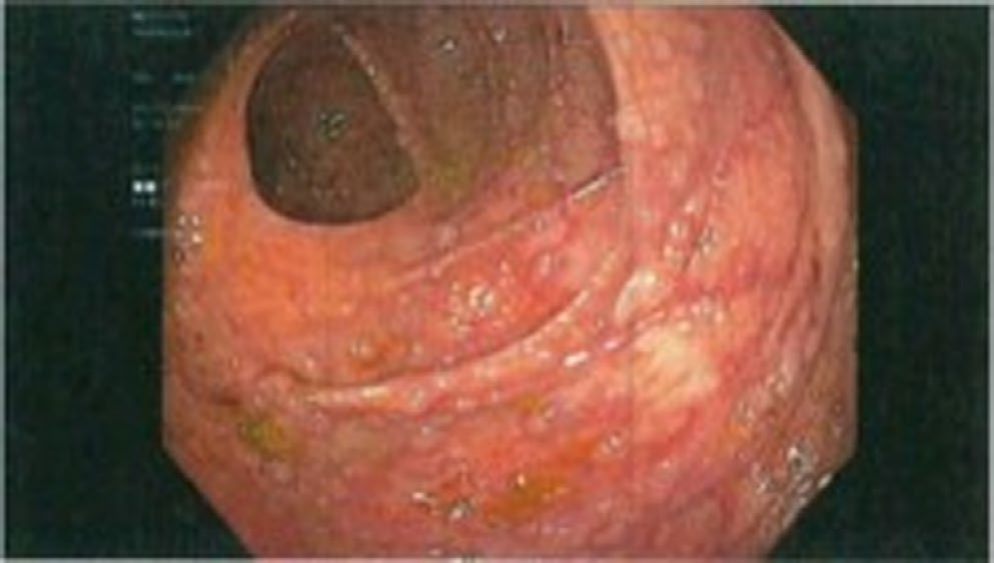
Colonoscopic picture of cecum

**Figure 4:**
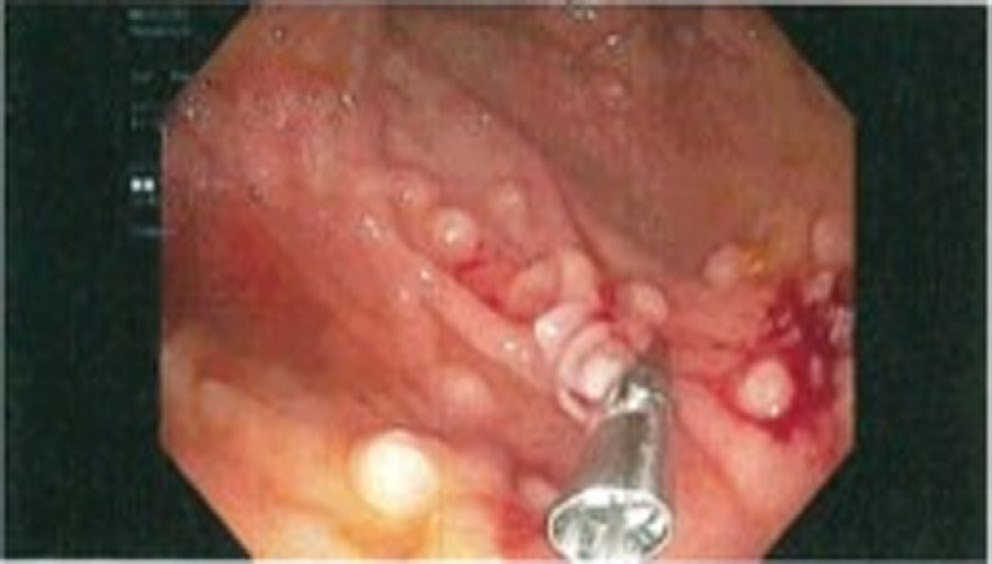
Cecum status postbiopsy and deployment of argon clip

**Figure 5:**
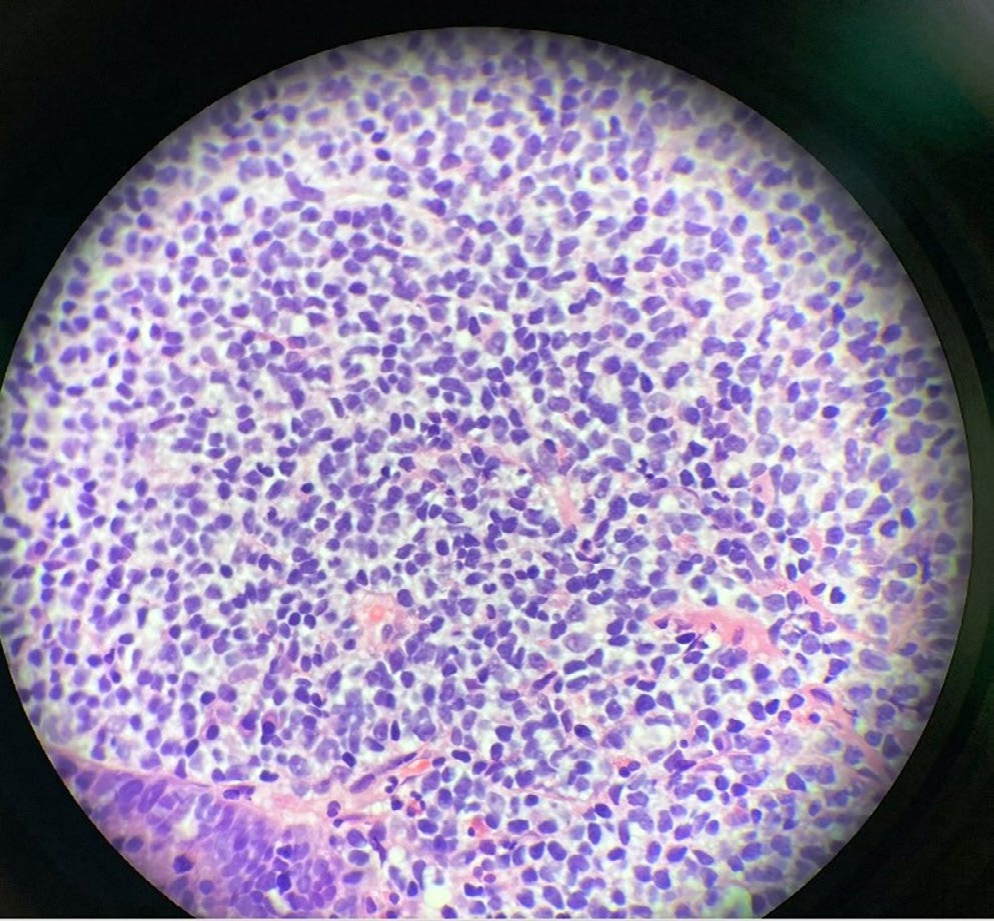
Biopsy from cecum on high magnification

Due to the precarious location, biopsy was not performed at that time. There were no signs of ulceration or any mucosal involvement. Some polyps were observed within the esophagus, but the stomach and duodenum were unremarkable. Colonoscopy revealed numerous polyps increasing in quantity from the transverse to the right colon. On colonoscopic inspection, polyps were uniform in size and shape resembling a polyposis syndrome. These are depicted in Figures 3 and 4. Multiple biopsies were obtained in the cecum, and ascending and transverse colon and then sent to pathology. Initial pathology report returned with the polyps as atypical lymphocytic infiltrate, which were later identified to be mantle cell lymphoma with increased uptake of Ki‐67 and positive fluorescence in situ hybridization (FISH) translocation with chromosomes 11 and 14. High magnification of cecal biopsy slide is located in Figure 5.

**Figure 6:**
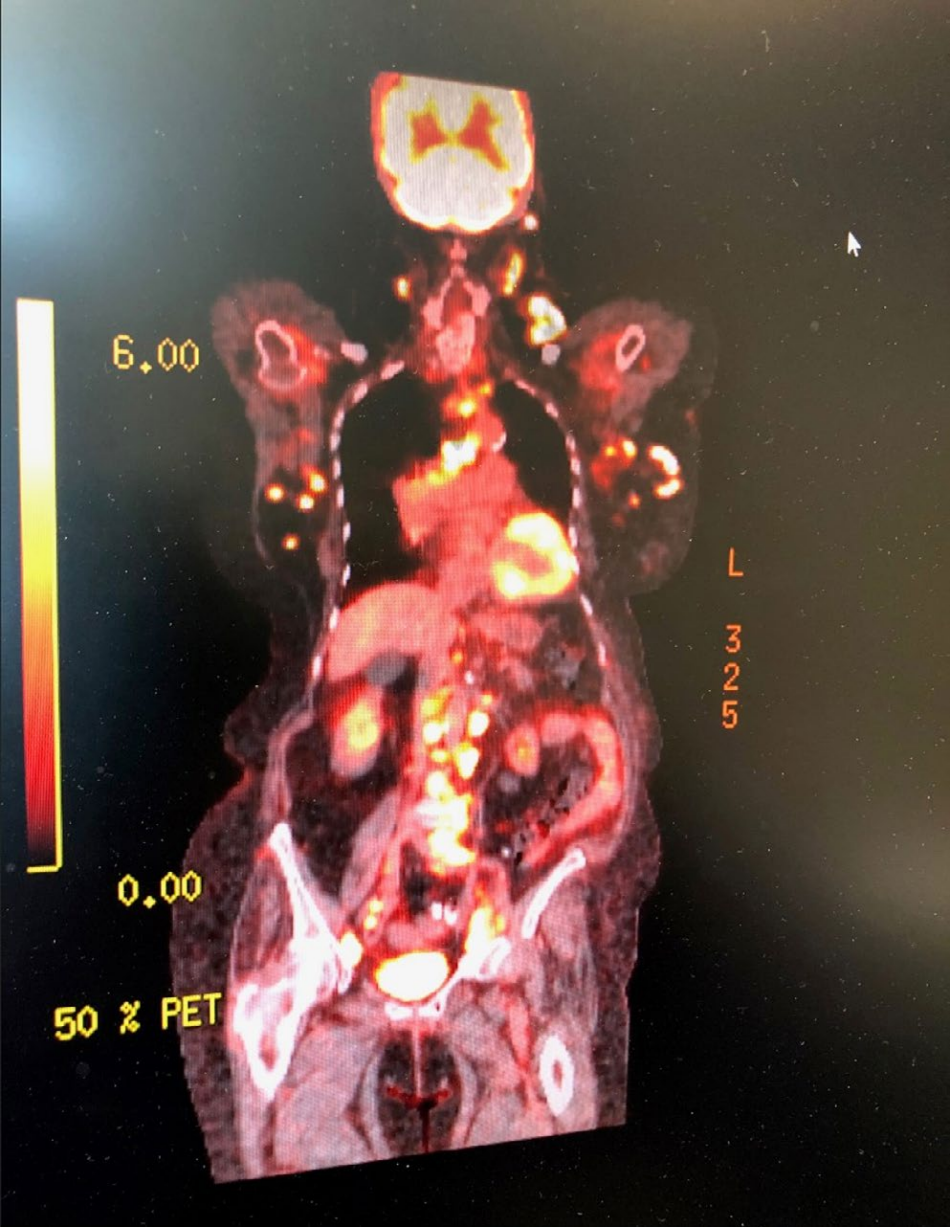
PET scan revealing uptake in Virchow's node, paraesophageal, axillary, paraortic, internal iliac, and inguinal lymph nodes

**Figure 7:**
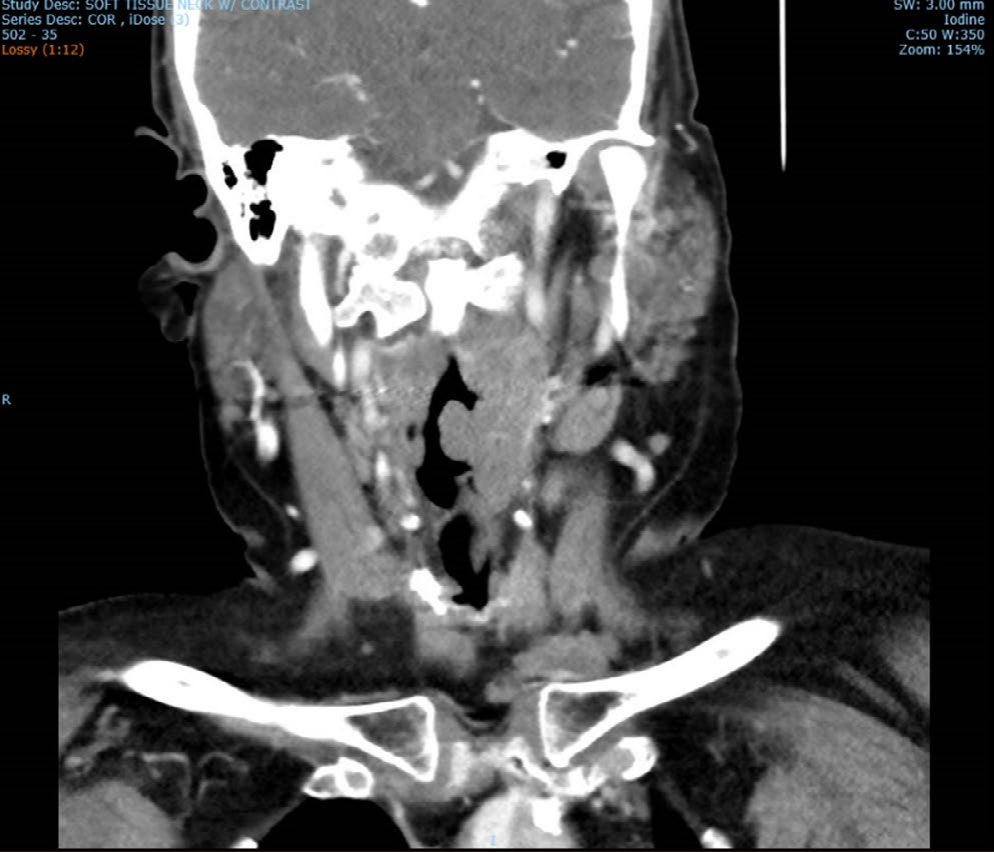
CT soft tissue coronal of laryngeal lesion

**Figure 8:**
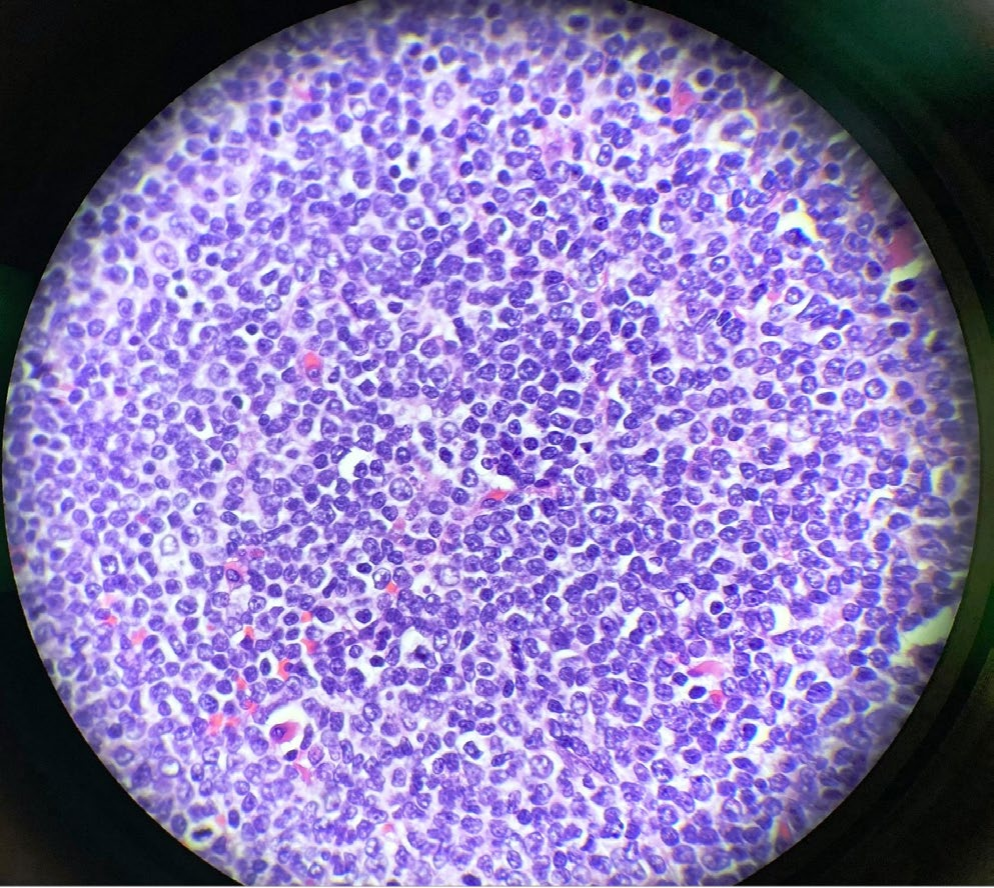
Biopsy from cervical lymph node in high magnification

Computed tomography (CT) scan soft tissues in the neck, CT chest, abdomen, and pelvis, along with positron emission tomography (PET) scan were performed (Figure 6). CT neck results showed multiple left‐sided pathologic nodes along with mass effect in the left‐sided laryngeal region and supraglottic region (refer to Figure 7). Sclerotic lesion in the vertebral body of C4 concerns for metastasis. CT abdomen pelvis was remarkable for periaortic lymph nodes, the largest of which was 1.3 cm. Excisional biopsy of cervical 1.5 cm cervical lymph node located posterior to the sternocleidomastoid and deep to the platysma was dissected from the surrounding tissue and sent to pathology for analysis (refer to Figure 8). Provisional report identified the biopsy to exhibit CD5‐positive B‐cell non‐Hodgkin lymphoma, later confirmed to be mantle cell lymphoma compatible with FISH analysis of biopsies from the colon. Morphologic features, increased mitosis and high Ki‐67 proliferation index (80%), indicate aggressive variant with blastoid and pleomorphic features.

## 2 DISCUSSION

Upon initial examination, the patient exhibited Virchow's node that was suspicious for malignancy. Due to lymphatic anatomy gastric cancer, lymphoma, breast cancer, lung cancer, esophageal cancer, and pelvic and testicular cancers comprised the preliminary differential diagnosis. Dysphagia along with the Virchow's node solidified rationale for performing upper endoscopy. Patient request, absence of previous screening, and Virchow's node were primary reasons for performing colonoscopy. Findings from endoscopy and colonoscopy increased the probability of advanced‐stage lymphoma, which were later confirmed by biopsies.

Non‐Hodgkin lymphoma (NHL) comprises 4% of cancers in the United States reported by the American Cancer Society.^1^ Mantle cell lymphoma (MCL) accounts for 5%‐6% of non‐Hodgkin lymphomas.^2^ This B‐cell type of NHL is associated with a translocation of chromosomes 11 and 14, which results in a fusion of the cyclin D1 gene with the immunoglobulin heavy chain. Of those diagnosed with MCL, the preponderance has been Caucasian men over the age of 60. Akin to other subtypes of NHL, the disease commonly presents with prominent cervical or axillary lymphadenopathy.

Outside of the lymphatic system, MCL has a predilection for the spleen, bone marrow, and gastrointestinal (GI) tract. Within the collection of gastrointestinal presentations to MCL, 10% are discovered as multiple lymphomatous polyposis (MLP) or lymphomatous intestinal polyposis.^3^ Cornes et al detailed the finding in 1961 as “a diffuse proliferation of atypical lymphocytes presenting as multiple polyps in the GI tract.”^4^ Due to the resemblance of other disease processes, multiple biopsies from different polyps in different locations should be sampled and reviewed by pathology. Other disease processes that are categorized by multiple polyps in the colon include polypoid syndromes such as familial adenomatous polyposis, and Peutz‐Jeghers syndrome. Colitis cystica profunda, colon cancer, and lymphoma are other etiologies of polypod colons. Biopsy and subsequent identification of the malignancy are critical due to the aggressive potential of this lymphoma. At the time of diagnosis, 70% of patients have an advanced stage.^5^ According to the Ann Arbor Classification, this is defined as either stage III or stage IV. Stage III involves lymph node regions on both sides of diaphragm. Stage IV demonstrates diffuse involvement of one or more extra lymphatic organs.

In order to elucidate a more tailored prognosis, calculators and histological markers are used. MCL International Prognostic Index (MIPI) is one that estimates overall survival in patients. It is comprised of the patient's age, lactate dehydrogenase, the Eastern Cooperative Oncology Group (ECOG) performance status, white blood cell count, Ki‐67 penetration, and its respective cell proliferation index. MIPI classifies patients into low, intermediate, and high risk in terms of mortality. Low risk is defined as median survival not reached after median 32 months' follow‐up and 5‐year overall survival rate of 60%. Intermediate risk is defined as median survival of 51 months, and high risk is defined as a median survival of 29 months.^6^ Although not perfect, this tool can aid physicians and patients in pursuing treatment options that align with personal goals of care.

In this report, we describe an uncommon case of stage III MCL presenting as MLP involving the transverse and ascending colon and cecum. This was performed after initial physical examination, which revealed Virchow's node (Troisier's sign). Remarkable CT findings lead to a cervical lymph node biopsy with histopathology results compatible with specimen findings from the colon. Other than dysphagia, our patient had no gastrointestinal symptoms. The patient was referred to an outside institution for treatment, and her outcome is unfortunately unknown. The aim of the case report is to increase awareness of MLP as a manifestation of MCL, and to encourage it being incorporated into the differential diagnostic workup of polypoid syndromes.

## 3 CONCLUSION

Mantle cell lymphoma is an aggressive form of non‐Hodgkin lymphoma with a variety of presentations. We highlighted a case where this malignancy presented with positive physical examination and endoscopic findings. Encountering multiple lymphomatous polyposis prompted further investigation to determine the extent of the lymphoma and subsequently guide treatment.

## 4 LESSONS LEARNED

As a cancer with a poor prognosis, the expeditious detection and treatment of mantle cell lymphoma is of utmost importance. Multiple lymphomatous polyposis is an uncommon presentation of this malignancy, and when exhibited should prompt clinicians to incorporate mantle cell lymphoma into their differential workup.

## CONFLICT OF INTEREST

Dr Ali has no financial disclosures at the time of this publication. Ms Gallivan has no financial disclosures at the time of this publication.

## AUTHOR CONTRIBUTION

Dr Ali: conceived and recommended the patient for case report, and performed the physical examination, colonoscopy, and lymph node biopsy. Ms Gallivan: compiled data, performed literature reviews of mantle cell lymphoma, created layout, and composed the report. Dr Ali: supervised and reviewed the final product.

## STATEMENT OF ETHICAL APPROVAL

Case reports submitted for publication do not meet national policy for protection of human subjects’ definition of research. This criteria require an investigation that contributes to generalizable knowledge about a disease or condition. Case reports develop information for education. Case report was written retrospectively.
